# Conditioned Medium from Bone Marrow-Derived Mesenchymal Stem Cells Improves Recovery after Spinal Cord Injury in Rats: An Original Strategy to Avoid Cell Transplantation

**DOI:** 10.1371/journal.pone.0069515

**Published:** 2013-08-27

**Authors:** Dorothée Cantinieaux, Renaud Quertainmont, Silvia Blacher, Loïc Rossi, Thomas Wanet, Agnès Noël, Gary Brook, Jean Schoenen, Rachelle Franzen

**Affiliations:** 1 GIGA-Neuroscience, Axonal Regeneration and Cephalic Pain Unit, University of Liege, Liege, Belgium; 2 GIGA-Cancer, Laboratory of Biology of Tumour and Development, University of Liege, Liege, Belgium; 3 Department of Neuropathology, University of Aachen, Aachen, Germany; Rutgers - New Jersey Medical School, United States of America

## Abstract

Spinal cord injury triggers irreversible loss of motor and sensory functions. Numerous strategies aiming at repairing the injured spinal cord have been studied. Among them, the use of bone marrow-derived mesenchymal stem cells (BMSCs) is promising. Indeed, these cells possess interesting properties to modulate CNS environment and allow axon regeneration and functional recovery. Unfortunately, BMSC survival and differentiation within the host spinal cord remain poor, and these cells have been found to have various adverse effects when grafted in other pathological contexts. Moreover, paracrine-mediated actions have been proposed to explain the beneficial effects of BMSC transplantation after spinal cord injury. We thus decided to deliver BMSC-released factors to spinal cord injured rats and to study, in parallel, their properties *in vitro*. We show that, *in vitro*, BMSC-conditioned medium (BMSC-CM) protects neurons from apoptosis, activates macrophages and is pro-angiogenic. *In vivo*, BMSC-CM administered after spinal cord contusion improves motor recovery. Histological analysis confirms the pro-angiogenic action of BMSC-CM, as well as a tissue protection effect. Finally, the characterization of BMSC-CM by cytokine array and ELISA identified trophic factors as well as cytokines likely involved in the beneficial observed effects. In conclusion, our results support the paracrine-mediated mode of action of BMSCs and raise the possibility to develop a cell-free therapeutic approach.

## Introduction

Spinal cord injury (SCI) is characterized by the primary lesion, rapidly followed by a cascade of cellular and molecular events that trigger the development of the secondary lesion, known to be deleterious for axonal regeneration and functional recovery. This worsening of the primary lesion is characterized by inflammatory reactions [1], [2], free radical production [3], glutamate excitotoxicity [4], neuronal death and oligodendrocyte apoptosis [5]–[7]. With time, necrosis spreads to adjacent tissues and a cystic cavity appears [8]–[10]. Moreover, the initiated spontaneous axonal regeneration is repressed by the inhibitory environment composed of astroglial scar, and myelin-derived inhibitory molecules [11].

In this context, numerous experimental studies have been performed to improve functional recovery, focusing on various parameters: control of inflammation [12], [13], rescue of neural tissue [14], [15], stimulation of axonal regeneration by modulation of the lesioned environment [16]–[18] or promotion of remyelination [19], [20]. Among the developed strategies, cell transplantation aims at replacing lost cells or producing beneficial effects at the lesion site. Among them, olfactory ensheathing cells, schwann cells, macrophages, fibroblasts, neural stem cells or BMSCs have been transplanted in various spinal cord injured contexts [21]–[29].

BMSCs are adult stem cells easily isolated from the bone marrow. BMSC transplantations have been widely studied in the context of SCI, and have proven beneficial effects on various aspects: inflammation, apoptosis, axonal regrowth, angiogenesis, tissue sparing, astroglial scar, and motor recovery [21], [23], [24], [30]–[34]. Nevertheless, BMSC transplantations have some disadvantages: they do not persist within the host injured spinal cord after transplantation [35], [36], and have a limited ability to replace lost cells [23], [37], [38]; finally, when used in other pathological contexts, BMSC transplants can have various adverse effects [39]–[44].

It is well known that BMSCs secrete a large variety of molecules and many studies have shown beneficial impact of these BMSC-released factors in different models [45]–[48]. Thus, we decided to study the effects of molecules secreted by BMSCs in an adult rat SCI model, using rat BMSC-conditioned medium (BMSC-CM). This original strategy constitutes a multifactorial treatment that could act on many aspects of SCI physiopathology, avoiding all disadvantages and ethical problems related to the use of cells.

The aim of this study is to evaluate the effect of BMSC-CM on secondary processes involved in lesion extension after SCI. *In vitro* experiments were run in parallel to assess the potential beneficial properties of BMSC-CM on apoptosis, angiogenesis and inflammation.

## Materials and Methods

### 1. BMSC culture and BMSC-CM preparation

BMSCs were obtained from the bone marrow of femurs and tibias of adult Wistar rats and characterized as previously described [21]. After having pooled cells from different donors, BMSCs were expanded in DMEM (Invitrogen) containing 4.5 g/L glucose, 0.58 g/L L-glutamine and 0.11 g/L pyruvate, supplemented with 10% non-heat inactivated fetal bovine serum (FBS, Invitrogen) and 100 I.U./ml penicillin and 100 µg/ml streptomycin (Invitrogen). Medium was changed twice a week, and cells were passaged when 90% of confluence was reached.

BMSC identity is confirmed on the basis of morphological criteria, plastic adherence and specific surface antigen expression: CD90(+), CD271(+), CD45(−) and CD11b(−). Differentiation ability of BMSCs was also evaluated after induction using specific media as previously described [97]. Oil Red O and Alizarin Red S stainings (Sigma-Aldrich) were used to assess adipogenic and osteogenic differentiation.

BMSC-CM was generated as follows: 90% confluent, passage 2–4 BMSCs in T75 tissue culture flask, were washed 3 times with phosphate-buffered saline (PBS) and transferred to a serum-free DMEM culture medium without phenol red during 48 h. CM from different flasks were harvested and pooled. Then, CM were concentrated 40 times by centrifugation at 4000 g for 15 min at 13°C, using 10-kDa MW cut-off filter units (Millipore). We consistently obtained, from 10 ml starting volumes, filtrate volumes of about 250 µl. Filter units were used only one time to avoid membrane saturation. Concentrated CM were then sterilized on 0.22 µm filters (Millipore), and stored at −80°C until use. The mean protein concentration of BMSC-CM is of 1.2–1.5 mg/ml. BMSC-CM was divided into small aliquots (500 µl-1 ml) before freezing to avoid repeated freeze/thaw cycles. Each aliquot was visually inspected before use to verify absence of precipitate (meaning a possible loss of protein function). There was no difference in protein concentration between fresh and freezed CM. As additional control, we also tested the stability of BMSC-CM by keeping it for 7 days at 37°C before assessing its anti-apoptotic property using the protocol described below.

Serum-/Phenol red-free DMEM, centrifuged and filtered, was used as control medium.

### 2. Cerebellar granule neuron culture and apoptosis

CGNs were obtained from 4- to 7-day-old Wistar rats, and dissociated as previously described [98]. Cells were seeded at a density of 125,000 cells/well. A purity of 95% was obtained and confirmed by double GFAP/β3-tubulin immunostaining. Three to four hours after seeding, CGNs were treated overnight with one of the following serum free media to induce apoptosis: (1) CGN culture medium, (2) CGN culture medium +25% BMSC-CM, (3) CGN culture medium +50 ng/ml TNFα (Invitrogen), (4) CGN culture medium +50 ng/ml TNFα +25% BMSC-CM. This proportion of BMSC-CM was chosen because pilot experiments showed the same effect when 25% or 50% of BMSC-CM were used. 25% was thus the better compromise to obtain an effect without neither affecting neuronal survival nor wasting BMSC-CM. Cells were then fixed in buffered 4% paraformaldehyde (PFA) for 10 min. Apoptosis was evaluated with the TUNEL method, according to the manufacturer's protocol (Roche). Cells were then counterstained with DAPI and mounted on glass slides. Photomicrographs of 20 random fields per experimental condition were taken (Olympus AX70) at 40× magnification.

For each condition, the total number of apoptotic cells was reported to the total number of cells within the 20 fields. Experiment was repeated 3 or 4 times. The results are expressed as a mean apoptotic rate in percent.

### 3. Ex-vivo aortic ring assay

Rat aortic rings were cultured in three dimensional type-I collagen gels as described by Sounni *et al.* [99]. The following culture conditions were tested: 5% BMSC-CM, 5% control medium, 20 ng/ml rat VEGF with or without 1 µg/ml anti-rat VEGF (PeproTech). This proportion of BMSC-CM was chosen to limit medium dilution that could affect neovessel regrowth and because it already triggered a strong response. Aortic rings were cultured for 9 days at 37°C in 5% CO_2_. Photomicrographs were captured, with a Zeiss phase contrast microscope at 2.5× magnification (Axiovert 25). A minimum of 3 aortic explants were used per experimental condition and experience was repeated at least 3 times. Quantification was performed following the algorithm described elsewhere [100].

### 4. Macrophage culture and activation

Rat macrophage cell line (CRL2192) from ATCC was expanded in Ham's F-12K medium (Invitrogen) supplemented with 15% heat inactivated FBS. Cells were transferred to Petri dishes (Falcon) at a density of 2×10^5^ cells/ml in the presence of Ham's F-12K/FBS supplemented with: (1) 25% control medium, (2) Lipopolysaccharides (LPS)+Interferon-γ (IFNγ), (3) LPS+IFNγ+25% BMSC-CM and (4) LPS+IFNγ+IL-4+IL-13. LPS from Escherichia coli 0111∶B4 (Sigma-Aldrich) and IFNγ (Millipore) were respectively used at a final concentration of 10 and 2 ng/ml. IL-4 and IL-13 (R&D Systems) were used at a final concentration of 10 ng/ml each. Proportion of 25% BMSC-CM was chosen for the same reason as for apoptosis experiment. After 24 h at 37°C, adherent macrophages were detached and cells and supernatants were collected. Cells were counted to normalize the cytokine concentration. Supernatants were centrifuged at 200 g to eliminate cells and the cytokine contents were analysed by ELISA. Media without macrophages were used as controls to remove cytokines that could come from serum and/or BMSC-CM. Experiment was repeated 2 to 4 times.

### 5. ELISA and antibody array

ELISA were used to assess *i*) the amounts of NGF, BDNF, IL-1β, IL-6, TNFα and IL-10 in BMSC-CM, as well as *ii*) the amounts of IL-1β, IL-6, TNFα and IL-10 in the macrophage culture supernatants, according to the manufacturer's recommendations (R&D Systems). For macrophage supernatant, values were related to 100,000 cells. Samples were run in duplicate for each experiment, and macrophage experiment was repeated 2 or 4 times. For BMSC-CM, samples were run in duplicate for each experiment, and experiment was repeated 2 to 13 times, according to the factor. Final results are expressed as a mean concentration in cytokine.

In parallel, a biotin label-based rat antibody array (RayBiotech) coated with 90 antibodies was used to obtain a broad view of molecules present in BMSC-CM. Three conditioned media from passage 3 BMSC cultures were tested.

### 6. Animal model

A total of 40 adult female Wistar rats (200 g) from the Animal Facility of the University of Liege were used. Rats were treated in strict accordance with protocols approved by the Ethical Commission for animal use of the University of Liege, and all efforts were made to minimize suffering. Rats were housed in individual cages from 7 days before the experiment, in a 12 h light/dark cycle, and in a temperature-controlled environment with access to food and water *ad libitum*. Animals were randomly divided into 4 experimental groups: BMSC-CM treated and control animals, sacrificed 1 or 6 weeks after SCI. 10 animals were studied in each group.

### 7. Contusion injury and BMSC-CM/control medium administration

Contusion SCI was induced with the « Infinite Horizons Spinal Cord Impactor IH-0400 » from *Precision Systems and Instrumentation*, LLC, Version 5.0., USA. Rats were deeply anaesthetized by inhalation of a mixture O_2_ (N25) with 5% isoflurane, anaesthesia being maintained with 2–3% isoflurane during surgery (Forene, Abbott).

The skin was incised to expose the thoracic vertebral column and a T10 laminectomy was performed. After stabilization of the spinal column, contusion lesion was performed using a 250 kilodynes force of impact. Dura was opened at the level of the lesion and 10 µl of BMSC-CM or control medium were directly dropped with a Hamilton syringe onto the lesion. A mini-osmotic pump (model 1007D, ALZET, flow rate: 0.5 µl/h/7 days) filled with BMSC-CM or control medium and linked to a catheter was then implanted to deliver the treatment at the lesion site. Muscles and skin were sutured. After 1 week, rats allowed to survive for 42 days were briefly re-anaesthetized to remove the empty pump, in order to avoid inflammation. Bladder function was assessed every day and manually emptied until rats regained control.

### 8. Neurological function evaluation

Two behavioural tests were used after SCI to assess motor performances of rats: the Basso, Beattie and Bresnahan (BBB) open-field test [101] consisting of a 21 point rating scale, and the grid navigation test with a 5 point scale [102]. All rats were accustomed to both tests 1 week before SCI. Two independent experienced individuals carried out all evaluations on a blind basis.

### 9. Euthanasia and histological procedures

After a deep anaesthesia with intraperitoneal injection of pentobarbital (200 mg/kg, Nembutal, CEVA Santé Animale), rats were transcardially perfused with 300 ml cold saline containing 0.01% heparine (LEO Pharma), then with 500 ml cold buffered 4% PFA in 0.1 M PBS (pH 7.4), 1 or 6 weeks post-injury. Spinal cords were dissected out, post-fixed for 24 h in 4% PFA at 4°C and cryoprotected for 48 h in a 30% sucrose buffer at 4°C. Serial, longitudinal (20 µm thick) and transverse (25 µm) sections were performed on, respectively, 15 mm-long and 10 mm-long tissue blocks centred on the lesion site. 1 out of 5 transversal sections were preserved. Inflammation and angiogenesis were studied on rats sacrificed 1 week after SCI (N = 10/group), using respectively CD11b and RECA-1 (Rat Endothelial Cell Antigen) immunostainings, on 3 longitudinal sections/animal. Axonal regeneration, astroglial scar formation and cavitation were studied on rats sacrificed 6 weeks after SCI. Axonal regeneration and astrogliosis were evaluated by immunohistology using antibodies against Neurofilaments (NF) and Growth-associated protein 43 (GAP-43) for axons and Glial fibrillary acidic protein (GFAP) for astrocytes, on 3 longitudinal sections/animal (N = 4/group). Finally, cystic cavity extension was measured after Luxol-fast blue/periodic acid - Schiff/hematoxylin staining on 6 transverse sections/animal (N = 6/group).

The following antibodies were used: *Primary antibodies*: mouse anti-NF200 (1∶400, Sigma-Aldrich); rabbit anti-GAP-43 (1∶1000, Millipore); mouse anti-GFAP (1∶400, Sigma-Aldrich), mouse anti-CD11b (1∶1000, AbD Serotec); mouse anti-RECA-1 (1∶200, AbCam). *Secondary antibodies*: biotinylated horse anti-mouse IgG and biotinylated goat anti-rabbit IgG (1∶500, Vector Laboratories). For anti-NF/GAP-43/GFAP/CD11b/RECA-1, sections were processed as previously described [21], except fixation in acetone and incubation in 0.5% H_2_O_2_ for RECA-1 antibody.

### 10. Image analysis

#### 10.1. Cystic cavity

To quantify cavitation along the ventro-dorsal axis, 6 stained sections/rat (N = 6/group), covering a distance of 1500 µm on either side of the lesion epicentre, were analyzed. Each section was observed under microscope (Zeiss, AxioImager Z1) to measure the height of the ventro-dorsal extent of the cyst relative to the one of the spinal cord midline (Mercator Pro software, Explora Nova). The mean ratio cyst extent/spinal cord extent was calculated for each experimental group and expressed as percentages.

#### 10.2. Immunostaining quantification

To quantify GFAP, NF, GAP-43 and CD11b, 3 longitudinal sections per animal were immunostained for each antigen. Bright field images of successive fields at 5× magnification were captured to perform a full reconstruction of each section (Leica DM6000B). On these photomicrographs, the immunostained area was measured in 3 boxed areas of 3 mm^2^ placed as follows: one at the lesion epicentre, one more rostrally (1000 µm from the epicentre) and one more caudally (1000 µm from the epicentre). Using analySIS software (Olympus), reconstructed photomicrographs were transformed in grey scales. A threshold intensity of staining was applied. The immunostained surface was then measured in each of the 3 boxed areas. Data are presented as the averaged percentage of total stained areas (µm^2^)/boxed area (µm^2^). To quantify the blood vessel diameters, RECA-1 immunostained sections were used. 3 longitudinal sections per animal were used and 3 fields per section were randomly chosen at the lesion epicentre. Bright field images at 10× magnification were captured with Zeiss microscope (AxioImager Z1). On each field, diameter of 10 blood vessels was measured with analySIS software. The blood vessel mean diameter for both experimental groups is considered.

### 11. Statistical analysis

All data were expressed as means ± standard error of the mean (SEM). The improvement of motor recovery was analyzed by Generalized Linear Mixed Model (GLMM), which evaluates the global evolution of the two experimental groups. Other data were analyzed by unpaired Student's t-test. P-value (p)<0.05 was considered statistically significant. All the tests were conducted using Statistica software (StatSoft), except for neovessel growth in aortic ring model (Aphelion (Adcis) and Matlab 7.9 (MathWorks)).

## Results

### 1. Characterization of rat BMSCs

**Figure 1:**
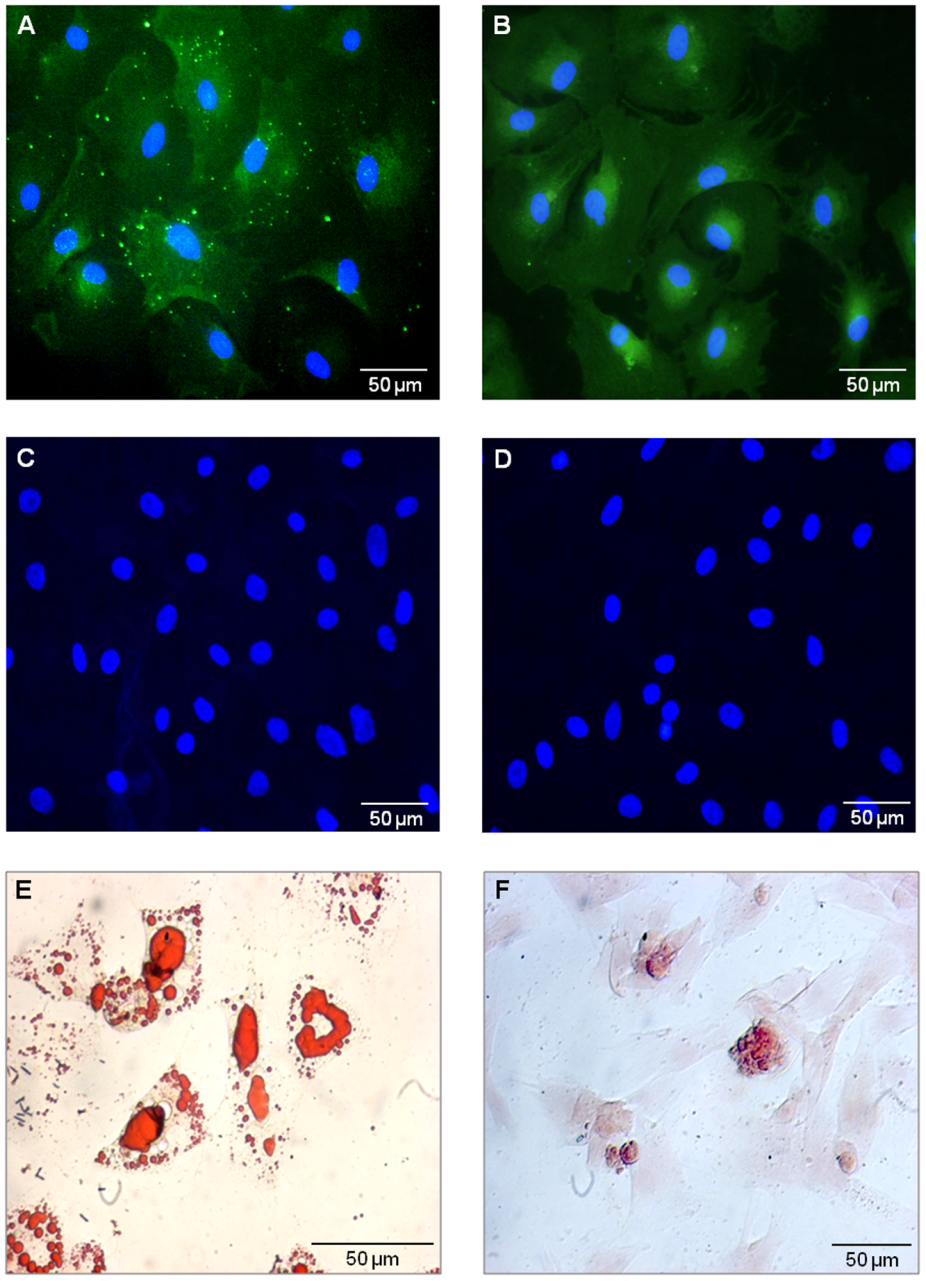
BMSC characterization. P4 BMSCs *in vitro* are immunostained for (**A**) CD90 and (**B**) CD271 (p75NGFr), but are negative for (**C**) CD45 and (**D**) CD11b. Nuclei are labelled with Vectashield-DAPI mounting medium. (**E**) Adipogenic differentiation of P4 BMSC revealed with Oil Red O and (**F**) osteocyte differentiation revealed by Alizarin Red S.

BMSCs were expanded in plastic dishes in classical *in vitro* conditions. Cells were characterized by their plastic adherence, the expression of specific antigens and their multipotency was checked by their differentiation capacity. Cells were used at low passages P2, P3 or P4, to work with young cells and avoid senescence-associated effects progressively acquired with passages [49]. P4 BMSCs express surface antigens CD90 and CD271 (p75NGFr) but didn't express CD45 and CD11b. Under specific induction media, cells differentiated into adipocytes and osteocytes (figures 1A–F).

### 2. BMSC-CM protects neurons from apoptosis *in vitro*

**Figure 2:**
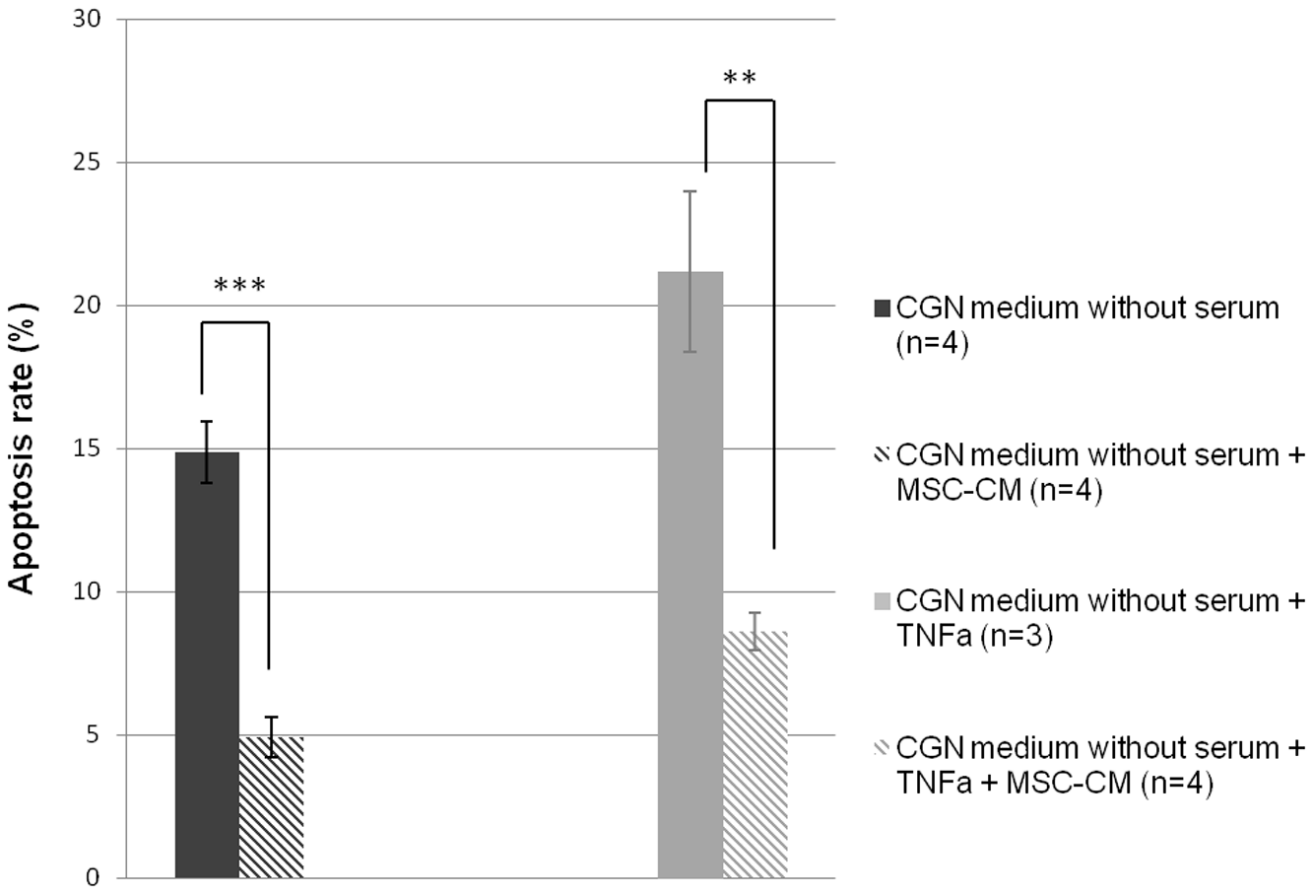
BMSC-CM protects neurons from apoptosis. Percentage of apoptotic CGN in serum-free medium or with TNFα (50 ng/ml), cultured with or without 25% BMSC-CM. BMSC-CM significantly decreases apoptosis. Experiment was repeated 3 to 4 times. *** p<0.001; ** p<0.01.

Cerebellar granule neurons (CGNs) were cultured and submitted to pro-apoptotic conditions: serum free medium or tumor necrosis factor alpha (TNFα) (50 ng/ml). BMSC-CM was added in each condition at a final proportion volume of 25%. After overnight treatment, cells were fixed and apoptosis rates evaluated using the Tunel method and a DAPI counterstain. Incubation with BMSC-CM significantly decreased apoptotic rates of neurons whatever the experimental condition (figure 2): in serum deprivation condition, BMSC-CM decreased apoptotic rate by 10% (from 14.8%±1.1 to 4.9%±0.7) (p = 0.0002, Student's *t*-test) and by more than 12% in TNFα-induced apoptosis (from 21.2%±2.8 to 8.6%±0.6) (p = 0.0038, Student's *t*-test). Results were identical with the use of BMSC-CM kept for 7 days at 37°C before performing the experiment (data not shown).

### 3. BMSC-CM is pro-angiogenic

**Figure 3:**
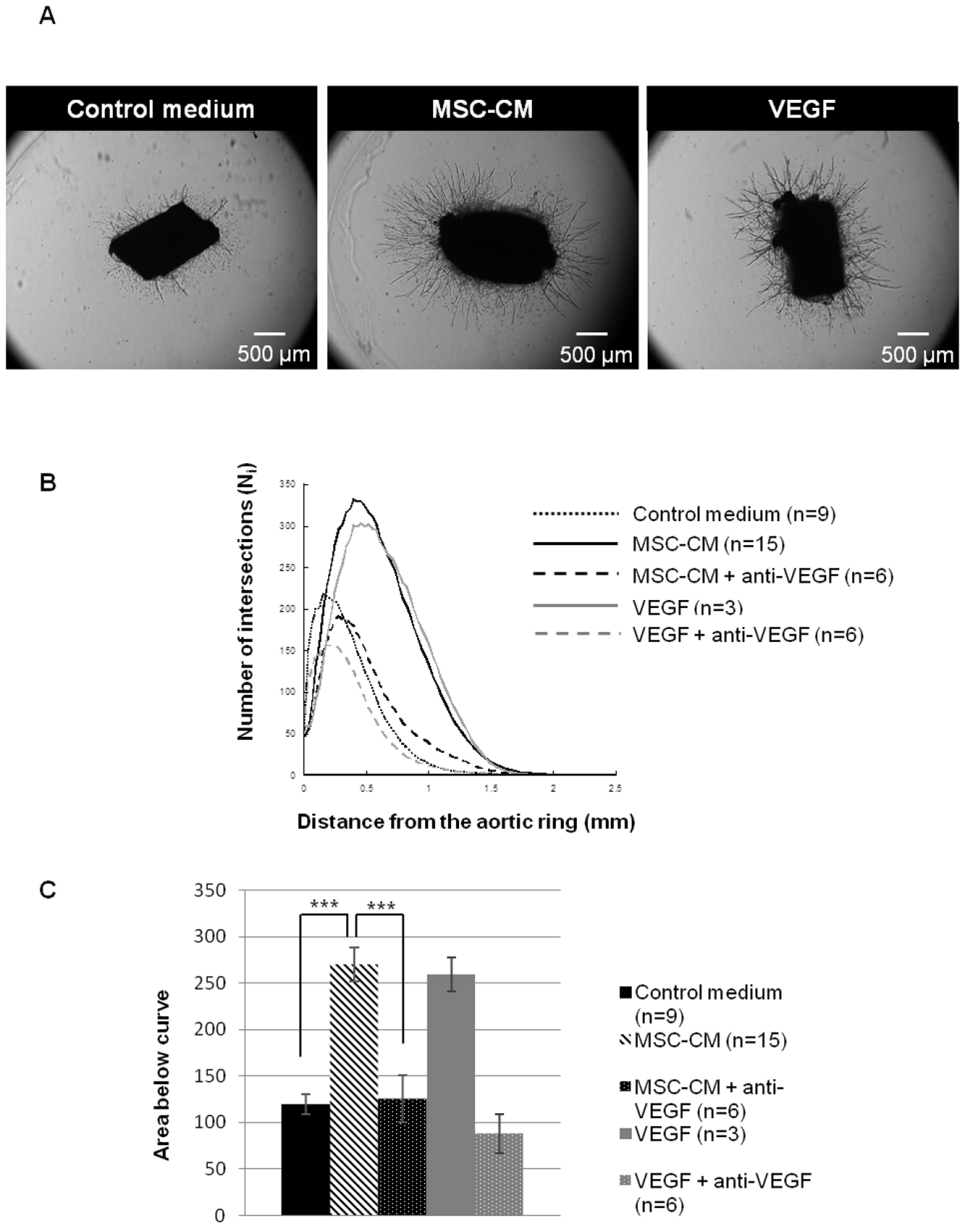
BMSC-CM promotes angiogenesis *in vitro*. (**A**) Representative photomicrographs of rat aortic rings after 9 days in culture medium containing 5% control medium, 5% BMSC-CM or 20 ng/ml of rat VEGF. (**B**) Quantification of number of intersections (N_i_) of neovessels treated with 5% control medium, 5% BMSC-CM and 20 ng/ml VEGF, with or without 1 µg/ml anti-VEGF. (**C**) Area below curves generated for each condition by the semi-assisted computerized quantification. Presence of BMSC-CM significantly favours angiogenesis and this effect is mediated by VEGF. Experiment was repeated 3 to 15 times. *** p<0.001.

As vessel growth favours axonal regrowth after SCI, we assessed the effect of BMSC-CM on angiogenesis. Rat aortic rings were incubated 9 days in a specific medium supplemented with 5% BMSC-CM, 5% control medium (negative control) or recombinant rat vascular endothelial growth factor (VEGF, 20 ng/ml; positive control) (figure 3A). Anti-VEGF functional antibody (1 µg/ml) was also added to assess a possible role of VEGF in observed effects. The number of intersections (N_i_) of neovessels as a function of the distance from the ring is illustrated on figure 3B. Areas below curves were calculated in order to include both parameters: N_i_ and the maximal distance from the ring reached by the neovessels. BMSC-CM induced a strong angiogenic response in comparison to the control (270.5±18.3 *vs* 119.3±11) to reach approximately the same level as with VEGF treatment. Anti-VEGF administration abolished the pro-angiogenic effect of BMSC-CM (270.5±18.3 *vs* 125.3±25.2) and, as expected, of VEGF. The differences were statistically significant between BMSC-CM and control medium (p<0.0001, Student's *t*-test) and between BMSC-CM and BMSC-CM+anti-VEGF (p = 0.0003, Student's *t*-test; figure 3C). This indicates that VEGF is involved in pro-angiogenic effects and must be present in BMSC-CM.

### 4. BMSC-CM favours a pro-inflammatory state of macrophages *in vitro*

**Figure 4:**
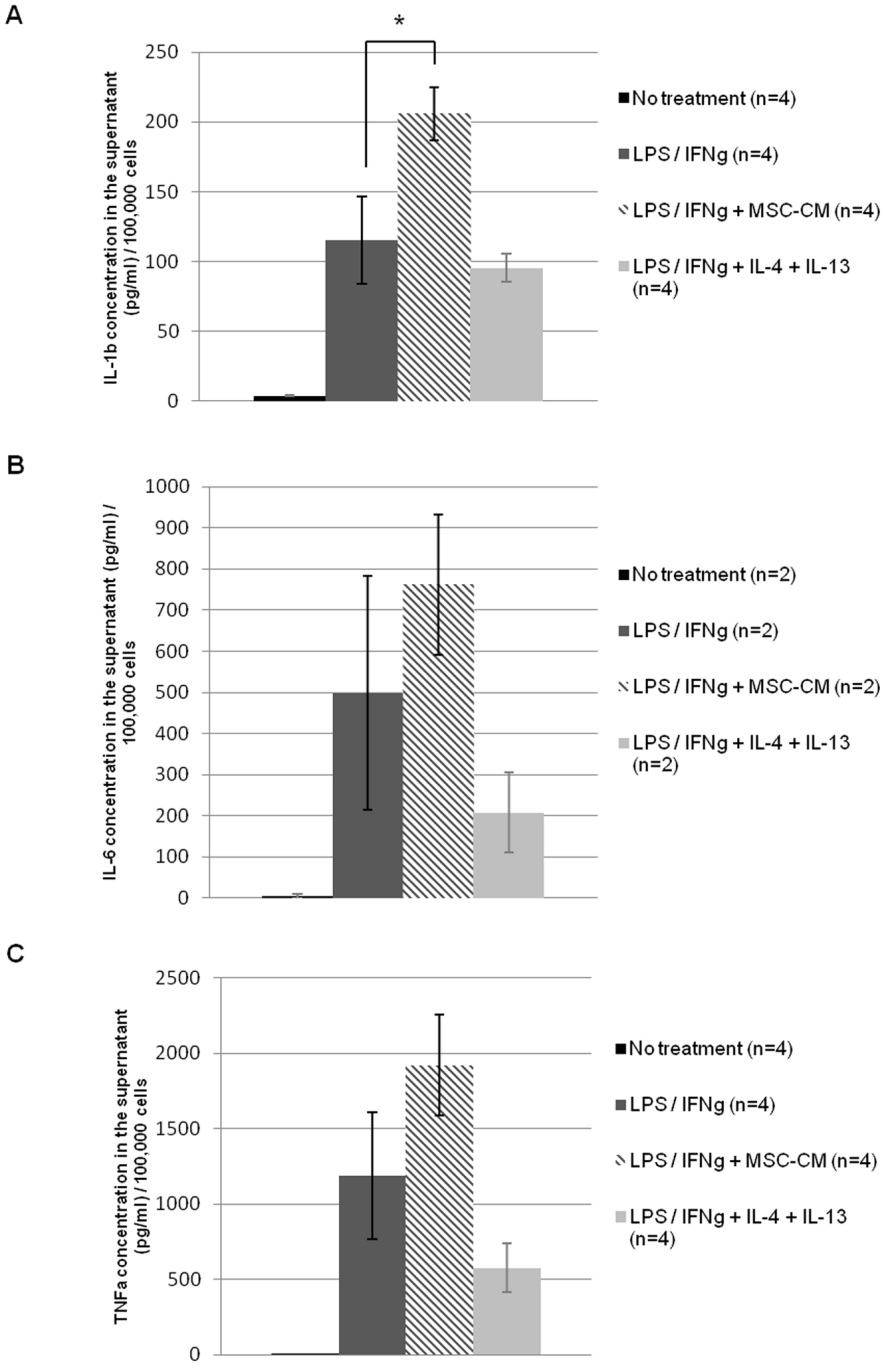
BMSC-CM stimulates macrophages to secrete pro-inflammatory cytokines. Concentration of IL-1β (**A**), IL-6 (**B**) and TNFα (**C**) in the supernatant of macrophages cultured in various conditions: non-activated, activated with LPS and IFNγ with or without 25% BMSC-CM, or with IL-4 and IL-13. BMSC-CM increases significantly IL-1β secretion by macrophages and tends also to increase their IL-6 and TNFα secretion. Experiment was repeated 2 to 4 times. * p<0.05.

In order to study the effects of BMSC-CM on macrophage phenotype, rat macrophages were activated with LPS (10 ng/ml) and IFNγ (2 ng/ml) to simulate the *in vivo* inflammation that follows SCI. Activated macrophages were further incubated with 25% control medium, 25% BMSC-CM, or anti-inflammatory cytokines: interleukin 4 and 13 (IL-4 and IL-13) (10 ng/ml). After 24 h, culture media were harvested to assess their content in IL-1β, IL-6, TNFα and IL-10 by ELISA. Each value was normalized for 100,000 cells, and the concentration of cytokines present in BMSC-CM or serum was deduced. Without LPS/IFNγ stimulation, 100,000 macrophages secreted 3.7 pg/ml±0.4 of IL-1β, while activated cells secreted 115.5 pg/ml±31.2. Incubation with BMSC-CM significantly increased IL-1β concentration in supernatant of activated macrophages from 115.5 pg/ml±31.2 to 206 pg/ml±19 (p = 0.048, Student's *t*-test). The use of IL-4 and IL-13 on activated macrophages reduced slightly the secretion of IL-1β to 95.6 pg/ml±10.2 (figure 4A). IL-6 and TNFα values also show a tendency to increase after BMSC-CM treatment, without reaching significant levels of differences (figures 4B–C). On the contrary, no secretion of IL-10 was observed (data not shown). Thus, BMSC-CM is globally pro-inflammatory on macrophages, as it exacerbates their secretion of pro-inflammatory cytokines.

### 5. BMSC-CM characterization

**Figure 5:**
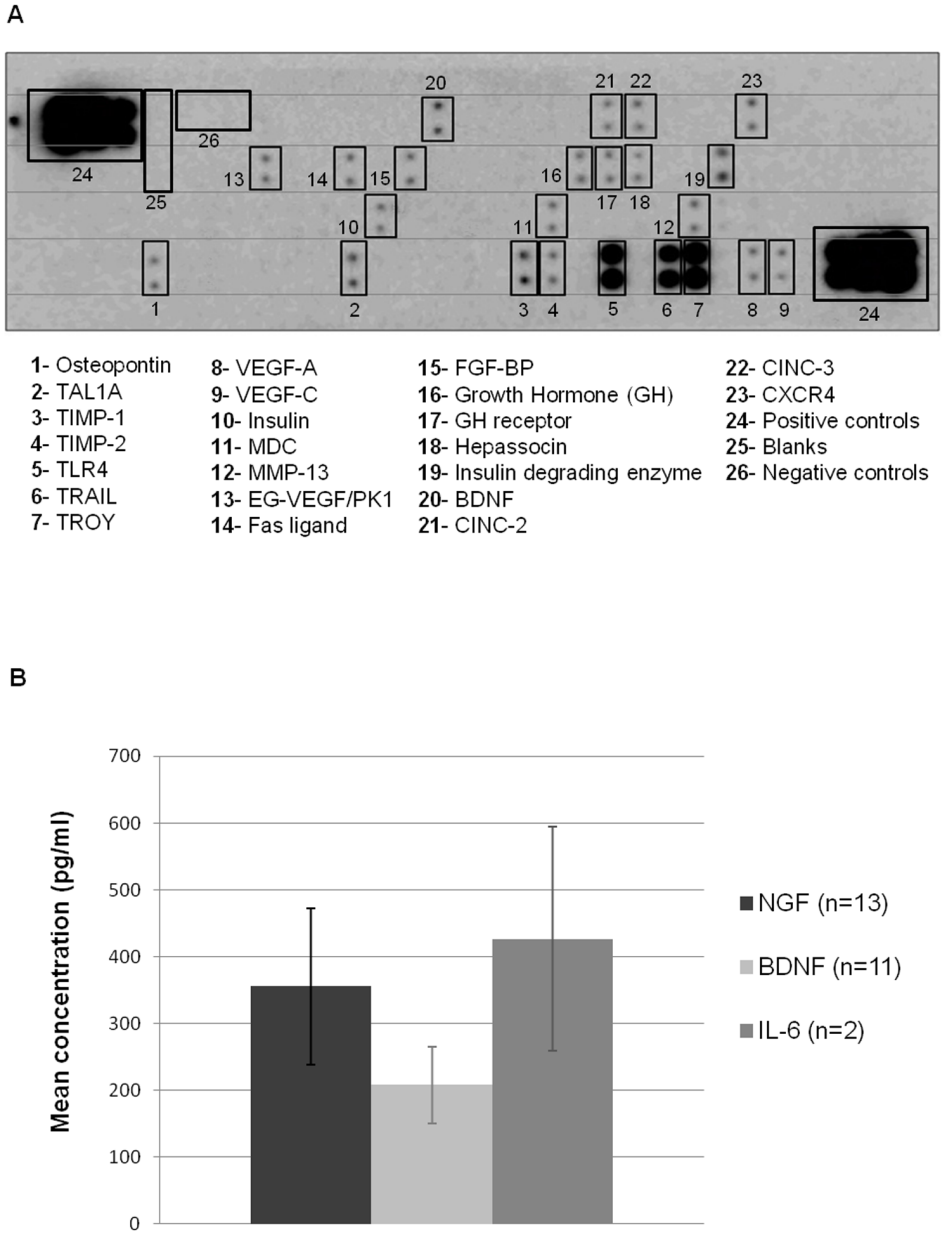
BMSC-CM characterization by antibody arrays and ELISA. (**A**) Multiple array membrane incubated with BMSC-CM reveals the presence of hormones, proteases, cytokines and trophic factors. Experiment was repeated 3 times. (**B**) Concentrations of NGF, BDNF and IL-6, measured by ELISA, within BMSC-CM. Experiment was repeated 2 to 13 times.

In order to further characterize BMSC-CM, we performed large antibody arrays (90 proteins) as well as ELISA assays. Antibody arrays, performed on 3 distinct BMSC-CM samples (figure 5A), reveal the presence of 23 factors involved in either apoptosis, inflammation, angiogenesis, oxidative stress, excitotoxicity, all involved in the modulation of the spinal cord injured environment (Table 1).

**Table 1:** Factors detected in BMSC-CM.

Phenomena	Specific properties	Factors
**Apoptosis**	Anti-apoptotic	VEGF-A, TIMP-1, CINC-3, TIMP-2, Osteopontin, Growth Hormone+GHR, EG-VEGF/PK1, TAL1A*, FGF-BP, BDNF
	Pro-apoptotic	TROY, TRAIL, Fas ligand/TNFSF6
**Inflammation**	Pro-inflammatory	TLR4, CINC-3, Osteopontin, EG-VEGF/PK1, CINC-2, MMP-13, MDC, Fas ligand/TNFSF6
	Immunomodulator	Osteopontin, CXCR4, MDC
**Angiogenesis**	Pro-angiogenic	VEGF-A, VEGF-C, Osteopontin, EG-VEGF/PK1, TAL1A*, MMP-13, FGF-BP
	Anti-angiogenic	TIMP-2
**Neuronal modulation**	Neuroprotective and Neuro-/neurito trophic	TIMP-1, CINC-3, Growth Hormone+GHR, BDNF
	Anti-neuritotrophic	TROY
	Neuronal differentiation	TIMP-2
	Modulation of neuronal activity	BDNF
**Stress**	Protection from excitotoxicity and oxidative stress	Osteopontin, FGF-BP
**Other**	Cell fate regulation	TIMP-1, TIMP-2, Hepassocin, Growth Hormone+GHR, EG-VEGF/PK1
	Cell survival	TIMP-1, TIMP-2, Growth Hormone+GHR, BDNF
	ECM modulation	TIMP-1, MMP-13
	Metabolism	Insulin degrading enzyme, Insulin, Growth Hormone+GHR

Antibody arrays against 90 proteins were performed on 3 different samples of BMSC-CM and identified 23 factors likely involved in the modulation of the spinal cord injured environment.

* protein not known to be secreted.

ELISA were performed on 4 cytokines: IL-6, IL-1β, TNFα, IL-10 and 2 neurotrophins: brain-derived neurotrophic factor (BDNF), nerve growth factor (NGF). The analysis of different BMSC-CM demonstrates that NGF and BDNF are present at 356 pg/ml±117 and 208 pg/ml±57 respectively, as well as IL-6 at 427 pg/ml±168 (figure 5B). IL-1β, TNFα and IL-10 were not detected in these samples.

### 6. BMSC-CM improves functional recovery after SCI

**Figure 6:**
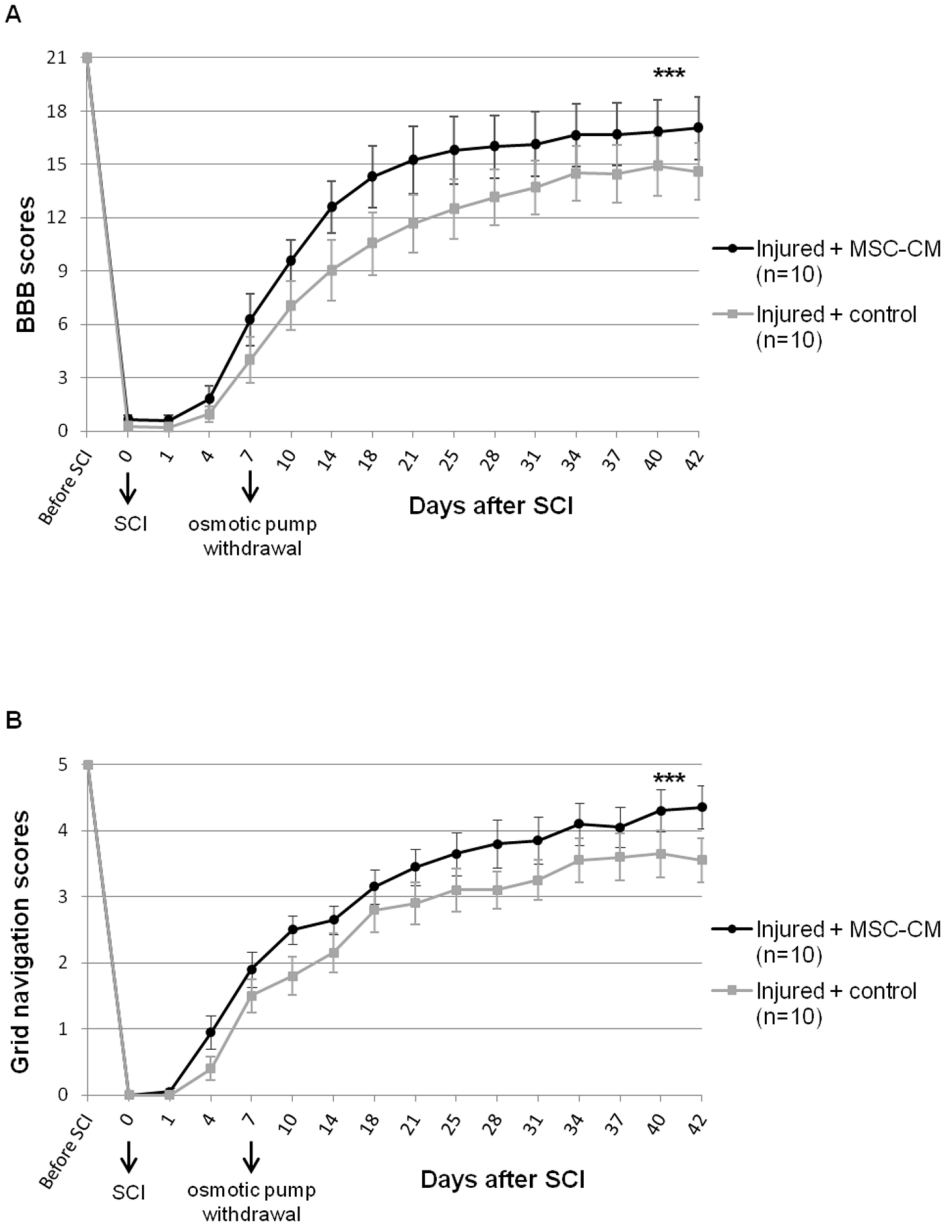
Behavioural analysis. Evolution of motor scores for BMSC-CM treated and control rats over 6 weeks, as assessed (**A**) by BBB scale and (**B**) by grid navigation scale. BMSC-CM treated rats obtained significantly higher scores compared to control animals. 10 animals per group were studied. *** p<0.001.

Spinal cord injured animals were submitted to 2 complementary behavioural tests. The Basso, Beattie and Bresnahan (BBB) open-field test was used to assess motor skills of rats until 6 weeks post-injury (figure 6A). The difference between treated and control groups was statistically significant (*Injured+BMSC-CM* vs *Injured+control* p = 0.0003, Generalized Linear Mixed Model). From day 4 after SCI, BMSC-CM treated animals show better scores than control animals and this difference was maintained during the study. Score 9 is crucial as it indicates the weight support step. The figure shows that this stage is reached by treated animals 4 days before control animals. Moreover, control animals never recovered their ability to separate toes and to maintain hindpaws in a parallel position at lift off during stepping (score of 16). These parameters, successfully performed by treated animals, bear witness to a fine recovery of motricity.

The grid navigation test was used to assess fine motor coordination and movement accuracy (figure 6B). Mean scores over time were significantly higher in BMSC-CM treated group compared to control group (*Injured+BMSC-CM* vs *Injured+control* p = 0.0001, Generalized Linear Mixed Model). We noticed that difference between both groups started from day 4 post-injury. Score 4, testifying a correct placement on grid bars, was reached only by BMSC-CM treated animals.

### 7. BMSC-CM reduces cystic cavity

**Figure 7:**
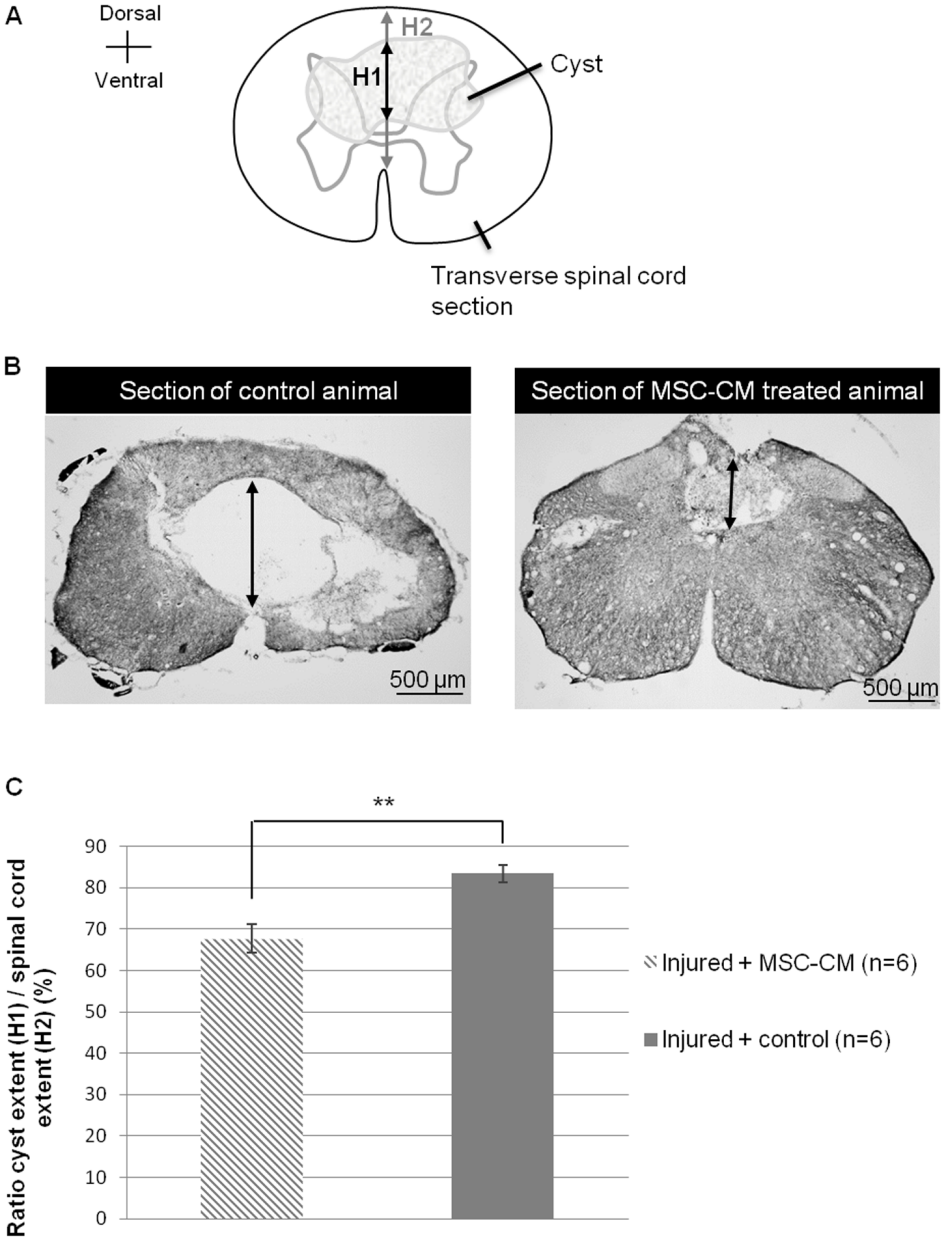
BMSC-CM reduces lesion extension. (**A**) Illustration of an injured spinal cord transversal section invaded by a cyst. For each section, cystic cavity extent (H1) and spinal cord extent (H2) were measured on the median axis. Both values were related to express a ratio in percents. (**B**) Photomicrographs of Luxol-fast-blue/periodic acid - Schiff/hematoxylin stained sections of BMSC-CM treated and control animals, taken at the lesion epicentre, 6 weeks after injury. Cyst extent is shown by an arrow. (**C**) Ratio cyst extent/spinal cord extent of BMSC-CM treated and control animals, 6 weeks after injury. BMSC-CM treated animals obtained a ratio significantly reduced compared to control group. 6 animals per group were studied. ** p<0.01.

The improved motor recovery observed in BMSC-CM treated animals could be explained by a reduced extension of the lesion. To investigate this aspect, spinal cord injured transversal sections from both experimental groups were performed 6 weeks after injury and stained with Luxol-fast-blue/periodic acid - Schiff/hematoxylin. Serial sections were then analyzed to quantify the ventro-dorsal extent of the cystic cavity (figures 7A–B). As spinal cord volume can vary over the lesion epicentre, cystic height was related to spinal cord height on the same dorso-ventral axis, and ratio expressed in percentages. BMSC-CM treated group shows reduced mean values compared to control group (*Injured+BMSC-CM*: 67.72%±3.4 *vs Injured+control*: 83.4%±2.1; figure 7C). Difference between BMSC-CM treated and control groups was statistically significant (p = 0.0029, Student's *t*-test).

### 8. BMSC-CM is pro-angiogenic *in vivo*

**Figure 8:**
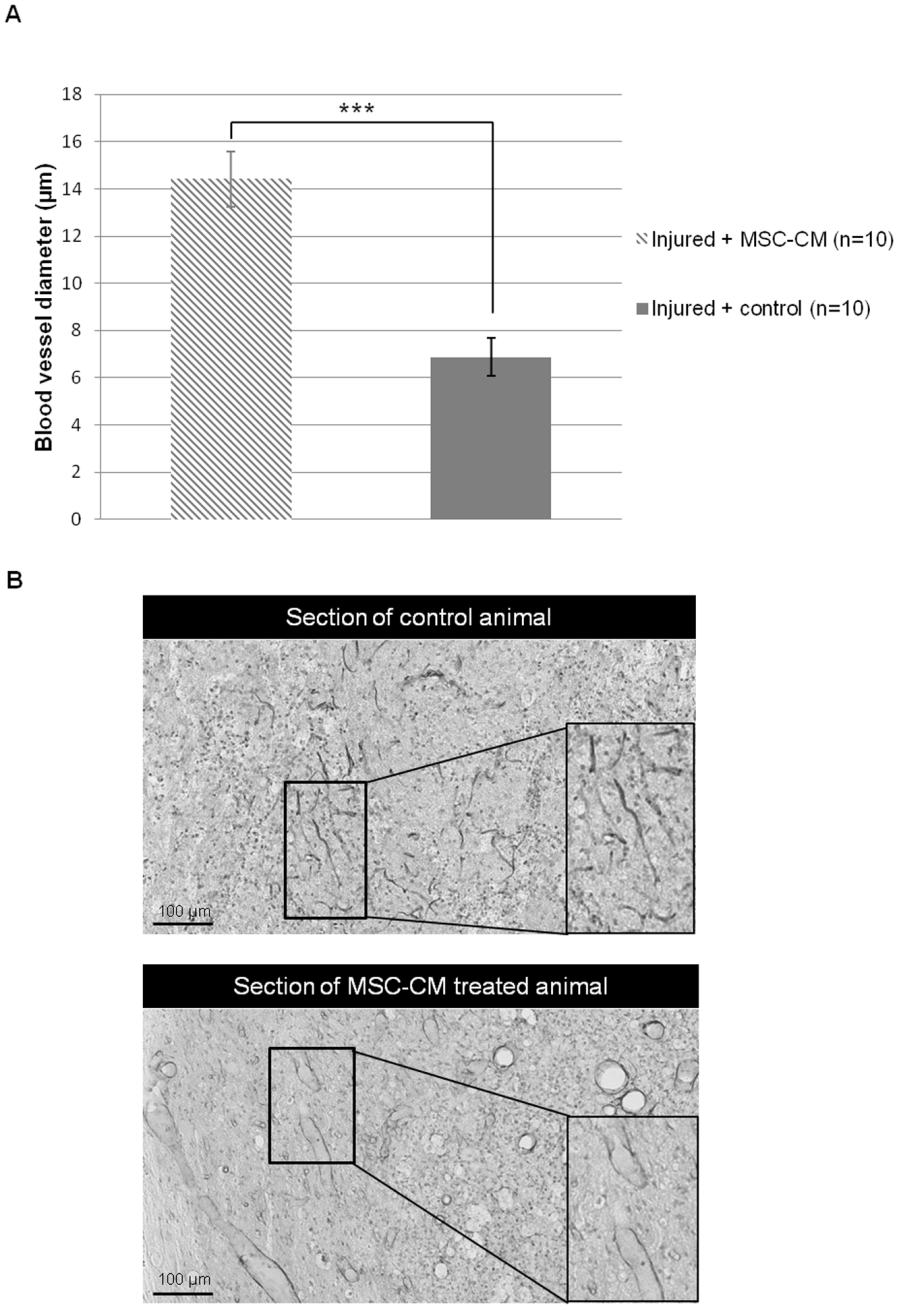
BMSC-CM increases blood vessel diameter within the lesion. (**A**) Mean blood vessel diameter at the lesion epicentre measured on RECA-1 immunostained sections from BMSC-CM and control groups 1 week after SCI, illustrated in (**B**). Blood vessels in BMSC-CM treated animals have larger diameters in comparison to control animals. 10 animals per group were studied. *** p<0.001.

Longitudinal spinal cord injured sections from treated and control groups were performed 1 week post-injury and immunostained for RECA-1. Diameters of blood vessels were measured at the lesion epicentre. Blood vessels exhibit larger diameters in BMSC-CM treated animals compared to controls (*Injured+BMSC-CM*: 14.4 µm±1.2 *vs Injured+control*: 6.9 µm±0.8; figures 8A–B). Difference between both groups was statistically significant (p<0.0001, Student's *t*-test).

### 9. *In vivo*, BMSC-CM has no effect on astrogliosis, macrophage invasion and axonal regrowth

To study astrogliosis, longitudinal sections performed 6 weeks after injury were immunostained for GFAP. Quantification of the immunostained surface was carried out in 3 defined areas. This value was related to the total area of each of the 3 zones to express a proportion of stained area. We didn't detect any difference between BMSC-CM treated and control animals (Student's *t*-test). After CD11b immunostaining, quantifications were performed as described above. Values obtained for BMSC-CM treated and control groups were not significantly different (Student's *t*-test). In order to assess the ability of BMSC-CM to favour axonal regrowth after SCI, anti-NF and anti-GAP-43 immunostainings were performed. Quantifications didn't reveal any difference between groups, whatever the delay (Student's *t*-test) (Table 2).

**Table 2:** Immunostaining quantifications.

Studied parameters	Studied regions	Proportion of stained area (%) in BMSC-CM treated animals	Proportion of stained area (%) in control animals	P-value and significance (treated *vs* control)	N/group
Astroglial scar (GFAP)	Epicentre	7.4±0.4	6.2±1	0.29 - NS	4
	Rostral	5.8±1.4	7.1±1.7	0.6 - NS	4
	Caudal	5.4±0.9	7.2±2	0.46 - NS	4
Inflammation (CD11b)	Epicentre	32.1±2	32.2±2.7	0.98 - NS	10
	Rostral	13.1±1.3	15±3.2	0.57 - NS	10
	Caudal	12.1±1.5	17±3.9	0.25 - NS	10
Axonal regrowth 1 week post SCI (GAP-43)	Epicentre	8.2±0.9	7.3±1.4	0.57 - NS	10
	Rostral	8±1	7.5±1.6	0.79 - NS	10
	Caudal	7.3±1.1	6.9±1.3	0.81 - NS	10
Axonal regrowth 6 weeks post SCI (GAP-43)	Epicentre	5.1±1.2	6.2±0.7	0.48 - NS	4
	Rostral	3.1±1.3	4.3±0.6	0.44 - NS	4
	Caudal	1.9±0.6	3.1±0.5	0.21 - NS	4
Axonal regrowth 6 weeks post SCI (NF)	Epicentre	5.4±1.6	6.3±1.4	0.71 - NS	4
	Rostral	6.9±2.5	4.8±1.3	0.48 - NS	4
	Caudal	8.9±3.1	6.7±2	0.59 - NS	4

After specific immunostainings (GFAP, CD11b, GAP-43, NF), quantifications of proportion (%) of stained area were performed in 3 defined areas. No statistical differences between treated and control rats were found.

## Discussion

Our results show that, *in vitro*, BMSC-CM provides protection against neuronal apoptosis, is pro-angiogenic and confers a pro-inflammatory phenotype to macrophages. *In vivo*, we demonstrate for the first time that BMSC-released molecules are able to reduce cystic cavity size along the ventro-dorsal axis at the lesion epicentre, to favour large blood vessel growth and to improve locomotor recovery in a spinal cord contusion injury model.

Many studies suggest that beneficial effects obtained after BMSC transplantation in SCI were more likely due to paracrine actions than to effective integration and differentiation of the cells within the host tissue [21], [23], [36], [38], [50]–[53]. Our study confirms this hypothesis.

We decided to deliver factors secreted by BMSCs in the form of concentrated conditioned medium (CM), to optimize potential effects. We also chose to use a fraction of this BMSC-CM that contains factors of a molecular weight >10 kDa, as this fraction was earlier described as beneficial on myocardial infarct size, oxidative stress and apoptosis [54]. Moreover, we eliminated serum from the medium because of its poorly defined composition.

Two complementary behavioural tests were used to assess recovery of motor functions: BBB as a global recovery test, and grid navigation to evaluate more precisely fine motor movements. In both tests, control animals show spontaneous recovery after spinal cord contusion. The great spontaneous recovery obtained here can also be explained by the type of the lesion, which, despite the use of a 250 kdyn force, doesn't severely affect the ventral part of the spinal cord, thus preserving white matter tracts [55]. According to the literature, 200 kdyn already corresponds to a severe injury, but this parameter is also known to vary upon the animal weight or strain [56], [57]. Despite this fact, BMSC-CM treated rats obtained significantly higher scores in both tests compared to control animals. Difference between groups started from days 4 to 7, indicating a rapid effect after administration, as also described after BMSC transplantation in a model of spinal cord contusion injury [24]. Later on, BBB scores continue to improve, with treated rats displaying at each delay higher scores compared to controls. In their study, Namiki *et al.* showed an improvement of inclined plane scores one week after BDNF infusion by osmotic pump in a SCI clip model. However, this evolution stopped very early after the end of the BDNF treatment [58]. The prolonged effect that we obtain may be due to the combined action of several factors present in our CM, with maybe redundant molecules with variable bioavailabilities. It is also possible that the cocktail of administered factors favours the survival of endogenous cells preserving the tissue from further lesion extension.

In other studies, after BMSC transplantation in contusion or compression models of rat SCI, locomotor improvements were also observed during weeks following the graft, with BBB scores reaching similar levels. For example, 4 weeks after spinal contusion and BMSC graft, Karaoz and colleagues obtain a BBB score of 15 while our treated rats reach the score of 16 at the same delay. Also, 3 weeks after BMSC graft in a compression injury model, Quertainmont *et al.* show that rats reach a BBB score of 12 which is even lower to the score of 15 reached in the present study [21], [24]. This reinforces our hypothesis that BMSC-secreted factors are beneficial after SCI and that they improve functional recovery as do BMSC transplants.

Improved recovery can be a consequence of neural tissue protection, which is favoured by angiogenesis. Angiogenesis is indeed known to induce axonal regrowth via the supply of oxygen and nutritional factors that helps preserving injured spinal tissue from further degradation [59], [60]. Moreover, it has been shown that the pro-angiogenic VEGF molecule possesses neuroprotective properties [61]–[63]. This factor is present in BMSC-CM and our *in vitro* data on aortic rings confirm that it is involved in the pro-angiogenic effect. Other factors which are known to promote angiogenesis were also detected in BMSC-CM: osteopontin [64], [65], matrix metalloproteinase-13 (MMP-13) [66] and fibroblast growth factor-binding protein (FGF-BP) [67], [68]. *In vivo*, blood vessels observed at the lesion epicentre of treated animals, even if not higher in number, have larger diameters compared to controls, and could thus provide, via increased blood flow, more nutritional substances and oxygen to damaged tissue, contributing to tissue protection, as already described after thoraco-abdominal aneurysm in pigs [69]. Lu *et al.* have also measured this parameter and their data suggest that atorvastatin treatment elicits larger vascular diameter, thus contributing to enhanced regional blood flow perfusion and neuron rescue [70]. So, larger diameters of blood vessels seem to be associated with a better tissue perfusion, protecting neuronal cells from degeneration. Yet, we didn't observe increased axon regeneration in treated spinal cords compared to control ones. In the same context, Dray's study suggests that even if some axons benefit from vascular support to accelerate their growth, this support is only transient and limited in time [60].

Tissue protection could also be related to a reduced apoptosis rate within the injured cord. Our data show that BMSC-CM protects neurons from apoptosis *in vitro*. Neuronal death and apoptosis rapidly followed by oligodendrocyte apoptosis are parts of secondary processes making the lesion worse. BMSC transplantations have been successfully used to reduce apoptotic death in the context of SCI, and associated to a better motor recovery [32]. In this study, we demonstrate that BMSC-CM treated spinal cords have a reduced depth of cystic cavity, protecting white matter tracts. You *et al.* demonstrated a positive correlation between spared ventral white matter and the final BBB scores of rats [55]. Also, the rubrospinal tract in the dorsolateral part and the corticospinal tract located in the dorsal part of the spinal cord white matter in rats are particularly important for precise limb movements, and can be assessed by grid navigation test [71]. Based on our behavioural data, we can thus conclude that the better motor recovery observed in BMSC-CM treated animals is a direct consequence of improved tissue sparing.

Among factors identified in BMSC-CM by cytokine arrays and ELISA, some may also contribute to tissue preservation. NGF stimulates the survival of sympathetic and sensory neurons, while TIMP-1 (tissue inhibitor of metalloproteinase-1) and CINC-3 (cytokine-induced neutrophil chemoattractant-3) are neuroprotective [72], [73]. BDNF administration decreases apoptosis and demyelination in a spinal cord compression model [74] and reduces astroglial scar formation [75]. Also, other factors, thus not described here, are known to be secreted by rat BMSCs: IGF-1, HGF, TGF-β1, EGF, SDF-1, MIP-1α/β, GM-CSF or FGF-2 [76], [77]. The fact that we didn't find those factors is first due to the fact that some of them were not included in our 90-protein array assay. Moreover, BMSC culture conditions may influence their secretome, which would explain why factors described in other studies were not detected here and conversely.

In our model, BMSC-CM doesn't affect astroglial reactivity. This result could be considered as unexpected, as both NGF and BDNF, present in BMSC-CM, are known to reduce reactive astrogliosis [75], [78]. This discrepancy is likely due to the variable concentrations of these two neurotrophins. Also, according to the literature, few studies report any effect of BMSC transplantation on astroglial scar development after SCI. This is also the case for axonal regeneration, which is rarely reported as associated to improved recovery after BMSC transplants [24], [79]–[82].

*In vitro* data on IFNγ/LPS-activated macrophages show that BMSC-CM further favours their pro-inflammatory state, as assessed by their significant increased IL-1β secretion and their obvious but non-significant increased IL-6 and TNFα production. In parallel, we also show that BMSC-CM contains IL-6, which possesses pro-inflammatory properties as well [83], [84]. *In vivo*, we didn't detect any difference between treated and control groups, in the number of macrophages that invaded the lesion site 1 week post-injury, as assessed by the total area stained for CD11b. Microglia/macrophages present within the lesion exhibited in both groups round cell bodies without branching processes, characteristic of “amoeboid” cells, and indicative of an activated status. This reactive cell form is associated with neuroprotective effects via trophic factor delivery and with phagocytosis that is essential to remove cell and myelin debris[23], [85]–[87]. In this context, we hypothesize that BMSC-CM has further favoured the activated state of resident microglia and invading macrophages, conferring a phenotype beneficial for tissue preservation, without affecting the number of inflammatory cells within the lesion. This hypothesis is in accordance with the fact that IL-1β and IL-6 do indirectly promote axonal outgrowth [88], [89] and protect neurons from death [90], [91]. In the literature, contradictory results have been described concerning the effect of BMSC transplantation on post-injury inflammatory reaction, going from reduced inflammation [92] to enhanced macrophage/microglia response [81]. Some authors also suggest that microglial activation after SCI is linked to the reduction of the lesion size [23], [93]. Indeed, microglia can be involved in neuroprotection via secretion of factors such as NGF, IGF-1 (insulin-like growth factor-1) or via up-regulation of FGF-2 (fibroblast growth factor-2) in neurons [94]–[96].

In conclusion, our data show that the use of BMSC-CM in the context of SCI is beneficial and not deleterious, and leads to improved motor recovery. This treatment constitutes a novel promising therapeutic perspective in SCI context. More investigations are needed to evaluate the potential of this treatment in chronic lesion models, and its future clinical application.
